# Novel experimental methods to investigate the effects of plant phytoliths on tooth enamel wear

**DOI:** 10.1098/rsif.2025.0175

**Published:** 2025-07-02

**Authors:** Yassmin Lakhal, Javier Redolat, Estíbaliz Sánchez-González, Paul Constantino, Michael A. Berthaume, Óscar Borrero-López, Elena Pinilla-Cienfuegos

**Affiliations:** ^1^ Nanophotonics Technology Center, Universitat Politècnica de València, Valencia, Spain; ^2^ Departamento de Ingeniería Mecánica, Energética, Energética y de los Materiales, Universidad de Extremadura, Badajoz, Spain; ^3^ Department of Biology, Saint Michael’s College, Colchester, VT, USA; ^4^ Department of Engineering, King’s College London, London, UK

**Keywords:** enamel, microwear, phytoliths, nanotechnology, micromechanics

## Abstract

Dental enamel is one of the strongest biomaterials found in nature, making its mechanical failure of significant interest to the biomaterials and dental communities. Recent studies on the mechanisms of enamel wear have yielded conflicting results, highlighting the need for more realistic experimental approaches. Here, we introduce a novel experimental methodology based on nanotechnology techniques and micromechanical/materials testing to simulate and characterize, for the first time, *in vitro* microwear caused by the sliding of artificial models of soft leaves containing phytolith particles against human dental enamel. While embedded phytoliths undergo mechanical degradation upon cyclic contacts, they increase the extent of pre-existing wear in enamel and decrease its mineral content. Surprisingly, the primary wear mechanism of enamel is ‘quasi-plastic’ (i.e. permanent) deformation enabled by failure of weak interphases, dominated at the microstructural scale. Mechanisms responsible for material removal in enamel at different length scales are identified and discussed. This research offers new insights into enamel failure that can further reveal information about an animal’s biology, behaviour, biomechanics and ecology, offering an interdisciplinary approach to the interface between the physical and life sciences.

## 1. Introduction

Natural dental enamel exhibits remarkable durability, surpassing that of many modern engineering materials relative to its hardness [1]. Consequently, teeth constitute a significant portion of the fossil record [2]. However, throughout their lifetime, teeth experience mechanical degradation, particularly in the form of fracture (sudden failure due to the unstable propagation of cracks driven by bite overloads) and wear (gradual, long-term material loss; [3]). Such damage is not necessarily catastrophic, and often, chipped and worn teeth can continue to function, albeit less efficiently—teeth are resilient [4,5]. The characteristic damage markings found on the surface of fossil teeth are highly useful for assessing animal feeding ecology (e.g. [6–12]).

The fracture and wear of human enamel have been extensively studied, including identification of the main mechanisms responsible for damage, critical fracture loads [3,13–15] and wear rates as a function of the chewing conditions [16], with attention to the role played by its microstructure. One aspect that has attracted considerable interest, but remains to be fully understood, is the effect of third-body, micrometric particles on enamel wear. Such particles may be extrinsic, present in the environment (e.g*.* dust [17]) or intrinsic in the food source itself (e.g. phytoliths [18]). While hard grits, like quartz particles, are generally accepted to cause abrasive wear to enamel [12,19–21], the effects of softer opaline particles, such as plant phytoliths, are less clear. Earlier experiments conducted using individual particles have sometimes yielded seemingly conflicting results [6,22,23]. A recent study employing a hard zirconia antagonist combined with a suspension of phytoliths (i.e. cumulative effect by many particles), at a relatively high contact load, concluded with statistical significance that, if the resolved load at individual particles exceeds a threshold value, phytoliths containing sharp edges can cause as much abrasion to enamel as quartz particles [24].

What is missing from previous studies are experimental methods capable of assessing the effects of plant phytoliths on tooth wear more realistically, particularly the cumulative effects of multiple phytoliths embedded in solid media of stiffness/hardness similar to that of actual plant leaves. This aspect is particularly challenging considering the techniques conventionally employed to conduct *in vitro* wear tests on enamel: adapted tribological (engineering, mechanical wear) tests and chewing simulators, which only permit the addition of a liquid/suspension to the test media. In addition, it is still unclear whether phytoliths or the enamel undergo any degradation other than fracture and wear during prolonged contact.

The present study aims to address some of the above issues by adopting a markedly multidisciplinary and multiscale approach. It develops a novel methodology, which combines nanotechnology techniques and micromechanical materials testing, to simulate *in vitro* microwear caused by the sliding of a synthetic, macroscopic leaf containing micrometre-sized phytoliths against human enamel specimens. Recognizing the challenge of fully capturing every structural detail of a natural leaf, this study presents a first attempt at developing a simplified—yet realistic—leaf model, aiming to clarify the role of plant phytoliths in tooth microwear. Damage mechanisms at different length scales are analysed employing a collection of microscopy and spectroscopy techniques in an effort to understand how macro-level actions induce micro- and nano-level structural changes in the enamel. The potential impacts of the results on anthropology and biology are briefly addressed.

## 2. Experimental procedure

A plant leaf model was created from a novel biocomposite consisting of a polydimethylsiloxane (PDMS)-based matrix with embedded phytoliths. Opaline phytoliths were obtained from wheat stems and leaves following a procedure described in detail elsewhere, which ensures that the physical properties of the particles are not modified [24]. Particle morphology was inspected by scanning electron microscopy (SEM; S-3600, Hitachi, Tokyo, Japan). PDMS, a silicone-based organic elastomer consisting of a repeating number of SiO(CH_3_)_2_ monomers, was used to make the polymer sheet. This polymer has a combination of properties, including transparency, non-toxicity, flexibility, low cost and ease of handling, that have led to its use as a substrate in countless applications [25]. The reported elastic modulus of plant leaves is between approximately 5 and 50 MPa [26,27]. The elastic modulus of PDMS is approximately 1−3 MPa [28], which is comparable to values in the low end of the former interval, typical of herbs and grasses [26]. Nevertheless, according to a simple rule of mixtures, the addition of stiffer phytolith particles will increase the modulus of the artificial leaves, bringing it fully in line with that of actual botanical leaves.

To obtain the phytolith–PDMS biocomposite, it was first necessary to mix homogeneously in a 10 : 1 ratio the pre-polymer and the crosslinking agent. The mixture was degassed by sonication for 20 min in an ultrasonic cleaner at room temperature to avoid pre-curing of the mixture, and then was carefully poured inside a flat glass mould. Hereafter, phytoliths were evenly dispersed over the top of the PDMS (around 10–20% of phytolith content). Finally, the mould was placed in an oven at 90°C for 45 min for the curing process of the elastomeric mixture. The PDMS biocomposites thus obtained were cut into small square pieces according to the required size.

**Figure 1.**
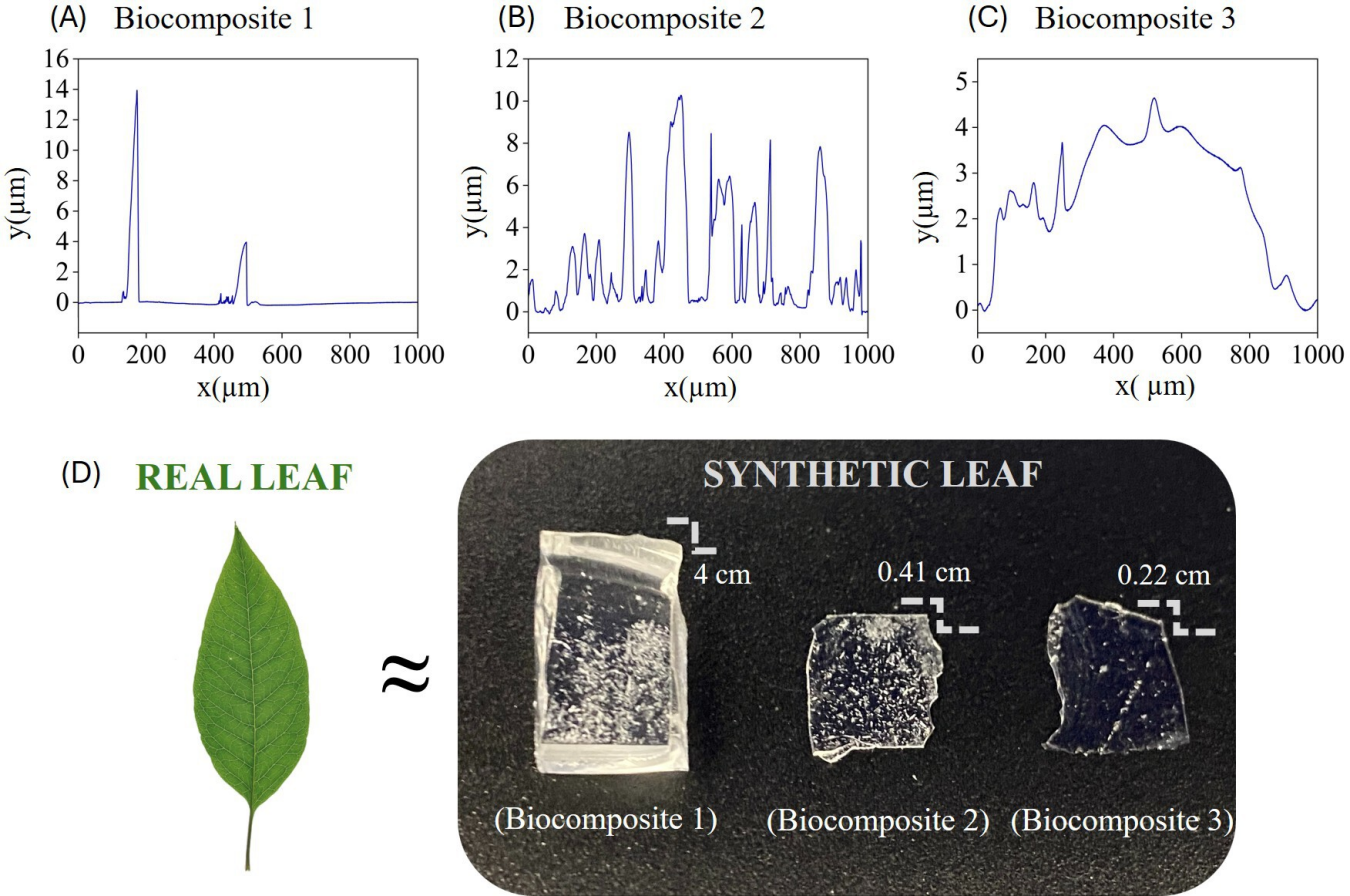
Profilometry measurements of biocomposite 1 (A), biocomposite 2 (B) and biocomposite 3 (C). (D) Comparison of a real leaf and a synthetic leaf. Images of the different biocomposites (artificial leaves) produced. Biocomposite 1: 4 cm thickness, with phytoliths dispersed after 20 min of the curing process. Biocomposite 2: 0.41 mm thickness, with phytoliths dispersed just before the curing process. Biocomposite 3: 0.22 mm thickness, with phytoliths dispersed after 10 min of the curing process.

An important parameter to consider during the biocomposite design, particularly in the precipitation phase, is ensuring that the phytoliths are not entirely covered by the PDMS, but rather, that part of their structure remains exposed on the biocomposite surface, yet adequately embedded to prevent detachment. This is crucial for successfully assessing the effects of phytoliths in the wear tests. To assess the degree of phytolith embedding in the prepared biocomposites and the degree of dispersion (separation among the phytoliths over the polymeric matrix), different time intervals for the addition of phytoliths during the polymer curing process were employed. Profilometry measurements were then conducted (Dektak 150 Surface Profiler), offering a rapid and non-invasive method to check the dispersion of phytoliths and the degree of their encapsulation (figure 1A*–*C).

Three types of samples of biocomposites were then produced with different preparation parameters: different thicknesses and various time intervals for the addition of phytoliths during the PDMS polymer curing process. Figure 1 shows an image of the three types of biocomposites: figure 1D (biocomposite 1) 4 cm thick biocomposite with phytoliths dispersed after 20 min of the curing process, figure 1D (biocomposite 2) 0.41 mm thick biocomposite with phytoliths dispersed just before the curing process and figure 1D (biocomposite 3) 0.22 mm with phytoliths dispersed after 10 min of the curing process. The first biocomposite leaf (biocomposite 1) exhibited a thickness significantly greater than that of an actual plant leaf (figure 1A). Moreover, upon conducting preliminary micromechanical tests, it was observed that the material’s elastic properties hindered the complete transfer of applied pressure, thereby resulting in pressure absorption. Notably, biocomposite 2 demonstrated an optimal distribution of phytoliths across its surface (as depicted in the profilometry profile in figure 1B, with a thickness of approximately 0.41 mm, closely approximating that of an actual botanical leaf. Biocomposite 3 presented a high degree of phytolith clustering (figure 1C). Based on these preliminary analyses, biocomposites type 2 were selected to perform the micromechanical tests over the enamel samples. Ensuring that the phytoliths are not entirely covered by the PDMS and remain partially exposed on the biocomposite surface was necessary for this initial phase of our study to directly assess the mechanical interaction between phytoliths and enamel. This approach simplifies the system by isolating the role of phytoliths in wear processes while guaranteeing they remain securely embedded to prevent detachment during testing.

Finally, it is important to note that the selected phytolith content (10−20%) was intentionally higher than the levels typically found in most plants consumed by herbivores. This elevated concentration was chosen to ensure sufficient damage on the enamel surface, facilitating its characterization through atomic force microscopy (AFM), SEM and Raman spectroscopy.

Enamel specimens were prepared from healthy third adult molars (impacted and semi-impacted wisdom teeth without any critical damage) provided by local dentists (CICOM, Centro de Implantología Cirugía Oral y Maxilofacial, Badajoz, Spain). Specifically, parallelepipedal specimens of thickness up to 1.5 mm were cut from occlusal surfaces, ground/lapped (30 μm) and finely polished using diamond suspensions of progressively decreasing grit size: 15 μm (10 min), 9 μm (10 min), 6 μm (10 min), 3 μm (15 min) and 1 μm (20 min). The amount of material removed from the tooth cusp during specimen preparation is at least 100 μm [29]. Preliminary microindentation tests [30] (applied load 200  g, using a Vickers tip) were conducted on the enamel specimens, and only those of hardness 3.5−4.5  GPa, characteristic of sound enamel, were selected for the study.

Enamel samples were preserved at low temperature, fully immersed in distilled water to prevent dehydration. Likewise, the phytolith samples were carefully preserved and manipulated to avoid structural changes.

Damage on the enamel specimens as well as on the artificial leaves was characterized at different length scales by optical (Zeiss optical microscope incorporated into the AFM/Raman system, Witec, Ulm, Germany), environmental scanning electron microscopy (ESEM; QUANTA 3D FEG, FEI Company, Hillsboro, OR) and AFM (alpha 300R, WITec, Ulm, Germany). In addition, micro-Raman spectroscopy measurements at room temperature (alpha 300R, WITec, Ulm, Germany) were performed. The confocal Raman imaging system was employed in the backscattering configuration using a 100× objective and a 600 grooves mm^−1^ grating with 3 cm^−1^ resolution, at an excitation wavelength of 532 nm and 25 mW power laser. The Raman spectra were then fitted using a Lorentzian function, extracting information on peak position and intensity. Raman images were acquired at 30 × 30 μm scan areas with 150 points per line and 150 lines per image with 0.01 s integration time per pixel.

Given its low depth of field, optical microscopy is limited to surface examination at relatively low magnifications (below 100×) in the bright field mode. Photo stitching of 100× magnification bright field images was employed to characterize large area imaging for the biocomposite and enamel specimens before and after the wear experiments. ESEM is used for finer surface observations, using secondary and backscattered electrons accelerated at low voltages, on uncoated specimens and without pumping high vacuum that could dehydrate the enamel. AFM and micro-Raman are used for high-resolution topographical inspections and chemical/mineral content analysis at the micro- and nano-scale, respectively.

**Figure 2.**
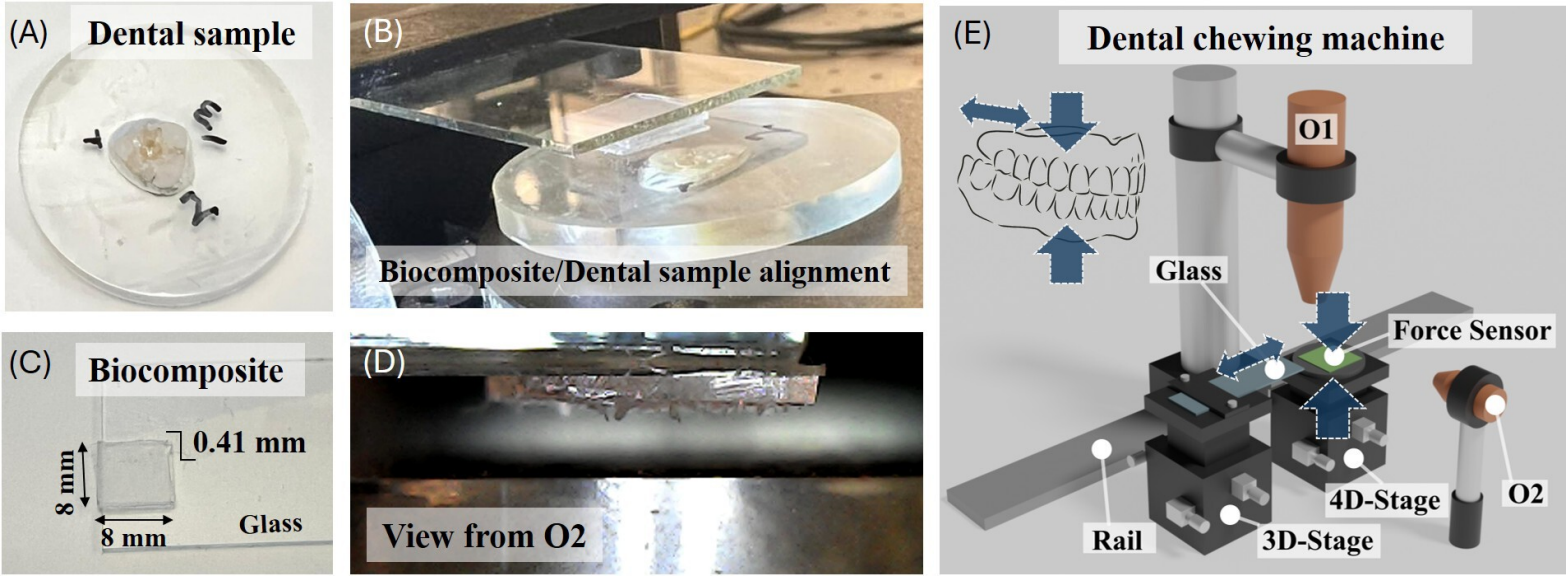
(A) Image of the dental sample. (B) Image of the biocomposite type 2 attached to the transparent mounting glass. (C) Lateral view of biocomposite/dental sample alignment. (D) Optical view from O2. (E) Schematic of the home-made experimental device developed for simulating chewing processes with artificial leaves. O1 and O2 refer to the imaging (optical) systems, which are USB-connected to a computer.

A home-made experimental device was developed for simulating chewing processes between the biocomposite or synthetic leaf and the dental sample (figure 2). The biocomposite is affixed to a glass holder (figure 2C). The system includes two aligned micropositioners. The first one features an XYZ movement platform (three-dimensional stage) that incorporates an accessory crafted using a three-dimensional printer to secure the sample holder system, enabling precise micrometric positioning of the biocomposite in all three axes and at the chosen position over the sample. The second micropositioner, a four-dimensional stage, offers movement in the three XYZ axes as well as rotation of the sample; the molar tooth (sample) is secured onto it. Displacements are controlled by micrometric screws and have a maximum travel distance of 13 mm with micrometric resolution. Regarding optical systems, the apparatus is equipped with a Navitar microscope (12×) with a Pixelink USB 3.0 camera positioned vertically (O1) and a digital optical microscope fitted with a stand to keep it fixed and oriented at 90° (O2) (figure 2D). Both are USB-connected to the computer for camera image visualization, facilitating proper alignment of the biocomposite/tooth system. The software used to visualize the system allows for image capture and video recording of the test. The lower load supported by the sample during the test is measured by a thin-film force-sensitive resistor sensor placed on top of the second micropositioner, just below the sample (figure 2E). The force sensor displays a square area of 5 × 5 mm and a load range from 0 to 10 kg. For the electronic design, an Arduino Nano based on the ATmega328P microcontroller is employed. This microcontroller enables analytical calculations, control of peripherals and data storage, among other tasks.

To perform the mechanical tests, the (tooth) sample was positioned onto the force sensor located in micropositioner 2. The synthetic leaf was placed on a transparent glass holder within the specifically designed accessory included in micropositioner 1 (figure 2C,D). The synthetic leaf was aligned and placed on top of the dental piece using the micrometric screws available on both micropositioners. For aligning and positioning the sample, optical access was available both vertically (with optical device O1) and horizontally (with optical device O2). Once the synthetic leaf was brought into contact with the sample, the desired pressure is applied onto the dental surface by approaching it to the leaf moving the vertical micrometer. The pressure is measured in real time on the force sensor. At this point, the test was conducted by sliding the dental piece for 10 cycles, each one of them of length (amplitude) 200 µm, at an average speed of 40 µm s^−1^, with an applied normal force of 30 N—within the range of masticatory forces in humans [31], as tests were performed on human enamel. Upon completion of the test, the leaf was withdrawn vertically. Tests were conducted on specimens without any damage and on specimens with previous wear damage. As control, an additional test was conducted using a particle-less PDMS leaf on pristine enamel. All tests were performed at room temperature and humidity without additional lubrication.

## 3. Results

**Figure 3.**
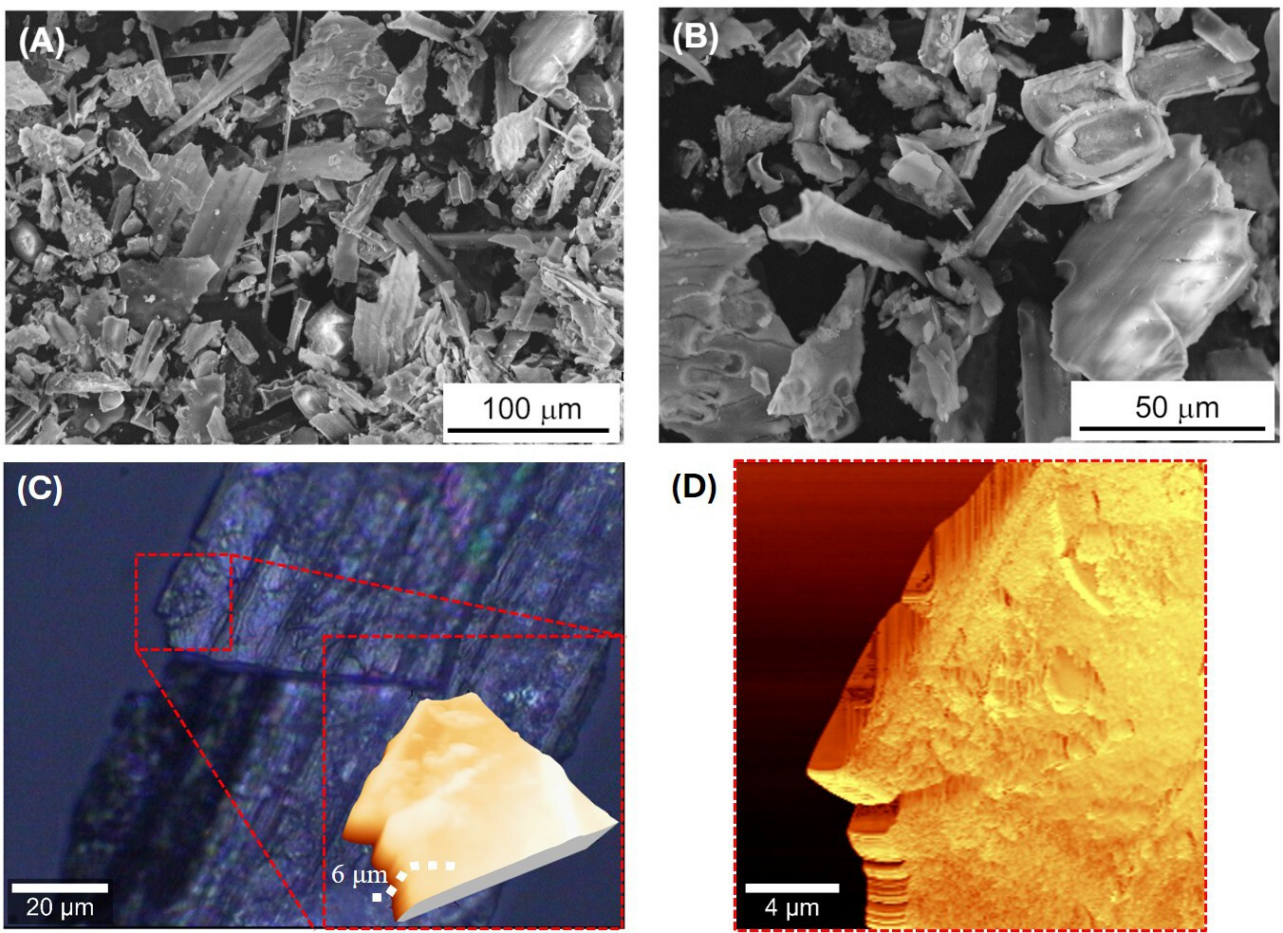
(A) SEM micrographs representative of the phytolith particles employed in this study, taken at low (A) and intermediate (B) magnification. (C) Optical bright field image 100x of a single phytolith. Inset: three-dimensional topographical AFM image of a part of the phytolith (highlighted in the optical image with a dashed red square) with a height profile of 6 μm, (D) AFM phase image of the phytolith image scan: 20 × 20 μm.

Figure 3 shows SEM micrographs representative of the plant phytoliths employed in this study. Figure 3A,B corresponds to as-received particles at different magnifications. Their sizes conform to a positively skewed normal distribution [24], with an average particle size *L*_ave_ of 24 μm (median 19 μm) and a standard deviation of 16 μm. Phytoliths appear to be relatively flat and elongated, with an average aspect ratio (*L/D*)_ave_ of 2.2 ± 1.3. Figure 3C corresponds to an optical bright field image at high magnification (100×) of a single phytolith. The inset shows a three-dimensional AFM topography image with a height profile of 6 μm. Importantly, AFM phase imaging (figure 3D) reveals that phytoliths often contain sharp edges, which could potentially cause enamel abrasion [10].

**Figure 4.**
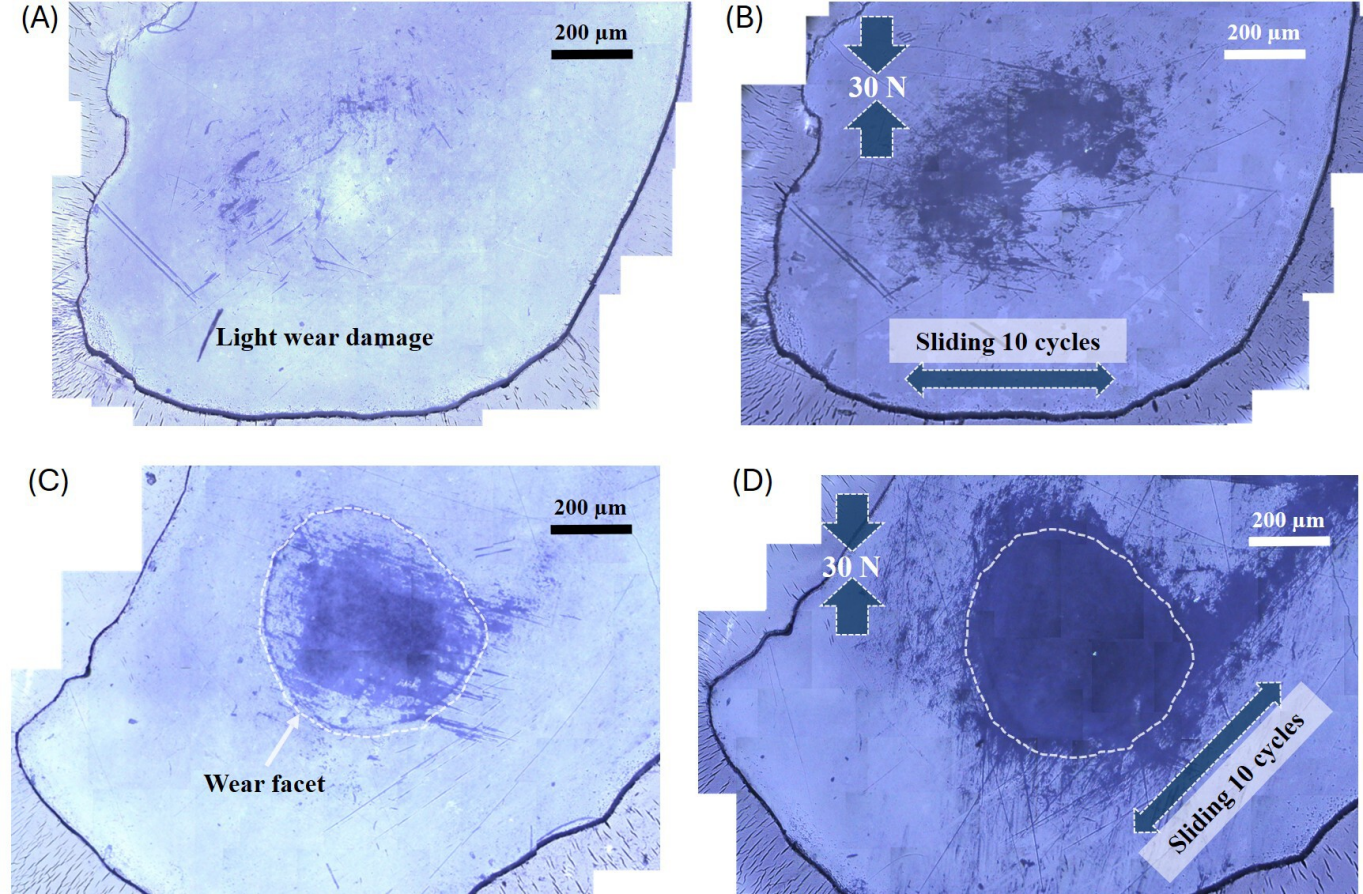
Optical macrographs of the surface (occlusal) of a polished dental enamel specimen (A) before; and (B) after a cyclic test against an artificial leaf. The mechanical test increases the extent of the light damage present on the enamel surface prior to testing. Optical macrographs of the surface (occlusal) of a polished dental enamel specimen (C) before and (D) after a cyclic test against an artificial leaf. The mechanical test increases the extent of the already severe damage present on the enamel surface prior to testing.

Figure 4 shows macrographs of the damage caused on select enamel specimens before and after the mechanical tests with artificial leaves. The images correspond to a photo stitching of 100× magnification optical images before and after the wear tests. Figure 4A corresponds to an initial enamel specimen (occlusal, polished surface) with a relatively low amount of wear. Figure 4B corresponds to the same enamel surface after a test with an artificial leaf (force 30 N, 10 sliding cycles). Clearly, sliding contact against the artificial leaf containing phytoliths increases the extent of the previous wear damage: it expands the boundaries and the depth of the wear scar, as well as the severity of the damage, with a greater density of microscratches on the test surface. Figure 4C shows an initial enamel specimen with a relatively large amount of occlusal wear, previously introduced *in vitro* in a controlled manner [16]. In this case, the abrasive effect of the phytolith particles becomes even more pronounced, with a greater increase in the damage that is observed after tests against an artificial leaf (figure 4D). The control test conducted using a particle-less PDMS leaf did not produce any detectable damage on the enamel surface (see electronic supplementary material, figure S2), thus confirming that the abrasion observed in figure 4 was caused by the phytoliths.

**Figure 5.**
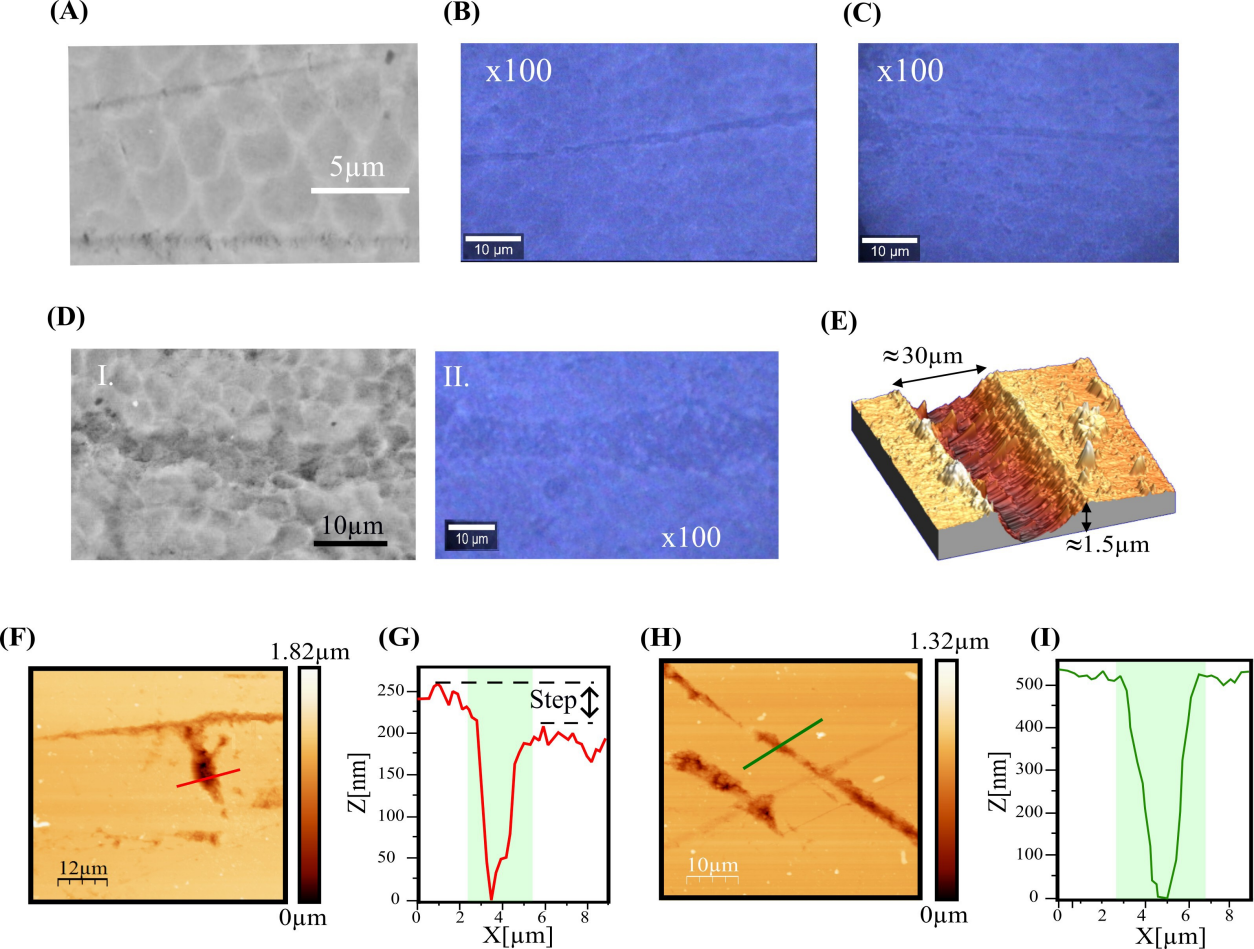
(A) High-magnification SEM micrograph at 5 µm scale, detailing the surface topology of a microscratch. (B) and (C) Optical microscopy images at 100× magnification, illustrating the morphology and extent of the scratches at a 10 µm scale. (D) Comparison of surface morphology: (i) high-resolution SEM micrograph showing detailed features of a scratched region and (ii) optical image at 100× magnification emphasizing structural variations in the enamel surface at 10 µm scale. (E) Three-dimensional AFM image of a wider mark. (F) AFM topographic image with corresponding profiles (G), where a step of 100 nm is highlighted with a black arrow. (H) The AFM topographic image of another similar microscratch with corresponding profiles (I).

Figure 5 shows details at different magnifications with SEM, optical and AFM images representative of the wear damage caused on the surface of pristine enamel specimens (i.e. free from previous wear damage) by the artificial leaves. Scratch marks with widths of the order of micrometres are observed, probably resulting from the phytoliths ploughing along as the artificial leaf slides. The primary damage mechanism identified is permanent/inelastic deformation at the particle/asperity level, leading to material removal (microwear) caused by the extrusion of plastically deformed material out of the microcontact [29]. This mechanism operates within individual enamel rods for the finer scratches (figure 5A–C), and both between and within rods for the coarser scratches (figure 5D (panels I and II)). The high-resolution AFM images (figure 5F–I) reveal that the microscratch marks have sharp cross-sectional profiles, with depths lower than 1 μm. The profiles generally do not show evidence of sink-in or pile-up formation at the edges, but in some instances contain steps of submicrometric height as indicated by black arrows in figure 5G. Three-dimensional details of wider marks reveal the presence of some microwear debris (figure 5E).

**Figure 6.**
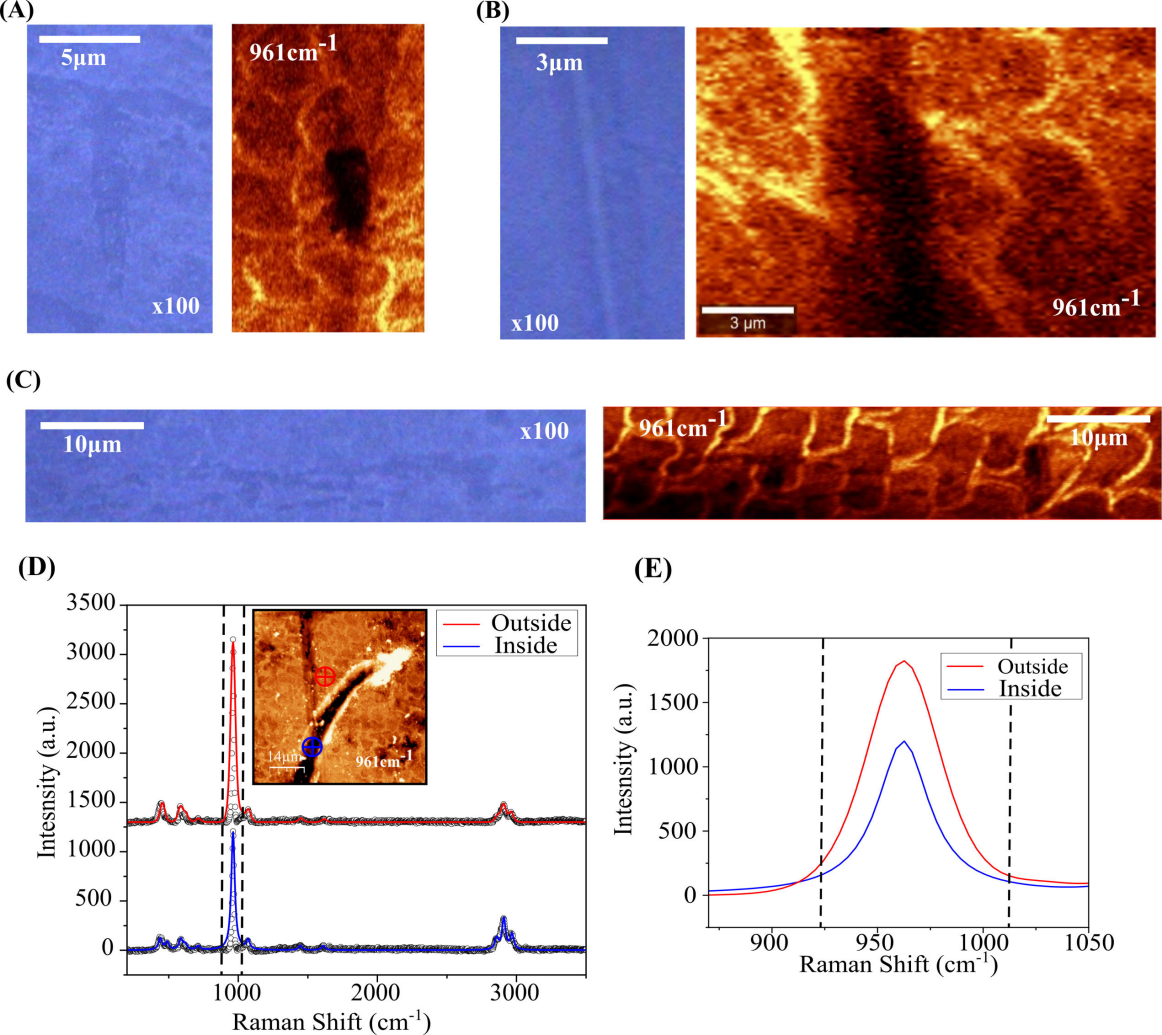
High-magnification details of a representative microwear marking, obtained by (A) 100× optical image (left) and Raman intensity map at 961 cm^−^¹ (right) of the same microscratch showing changes in the phosphate (PO₄) Raman vibrational mode corresponding to hydroxyapatite. (B) 100× optical image (left) and Raman image (right) of another microscratch. (C) Larger scale optical (left) and Raman (right) images reveal additional details of microscratch damage across a broader area. (D) Lorentzian peak fitting of the Raman spectra inside (blue) and outside (red) a thicker damage scar (inset shows Raman mapping with measurement positions). (E) A detailed view of the 961 cm^−^¹ Raman peak reveals significantly lower intensity inside the microscratch compared with outside, indicating both deformation and demineralization of the enamel.

Figure 6 reveals further details of the microwear damage caused by individual phytolith particles. Figure 6A (right panel) shows the Raman image obtained from changes of the intensity in the 961 cm^−1^ peak—corresponding to phosphate (PO_4_) Raman vibrational mode characteristic of the hydroxyapatite/mineral component of enamel [32]—for the same area of the microscratch depicted in the 100× magnification optical image (left panel). Figure 6B shows the same information from another microscratch. Figure 6B corresponds to larger scale optical (left) and Raman (right) images of another microscratch. The Raman image reveals how the abrasion induces permanent deformation of the enamel rod arrangement on the surface, at the microstructural scale. Figure 6E corresponds to the Lorentzian peak fitting of a Raman spectrum inside (blue line) and outside (red line) of a thicker damage scar (positions marked in the inset). A detail of the 961 cm^–1^ peak is presented in figure 6F right panel, where it can be clearly seen that the peak has a significantly lower intensity inside than outside. This indicates that, in addition to deformation, the abrasion process induces some degree of demineralization in the enamel [33].

**Figure 7.**
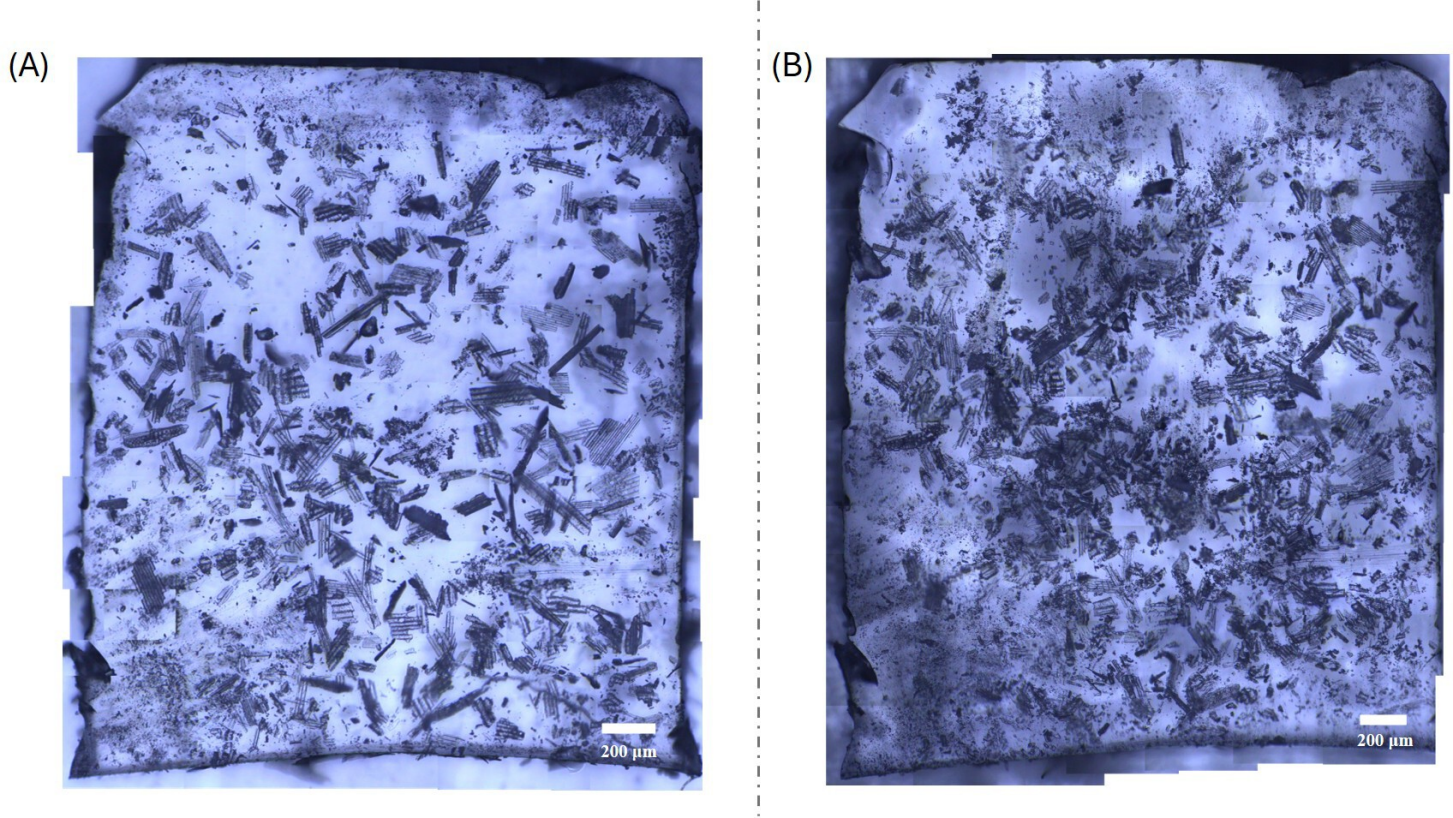
Optical macrographs of an artificial leaf: (A) before and (B) after mechanical testing against enamel. Phytoliths undergo significant mechanical degradation in the form of wear and fracture.

Figure 7 contains representative macrographs of an artificial leaf before (figure 7A) and after (figure 7B) sliding-contact testing against enamel. The images reveal that phytoliths undergo significant mechanical degradation during testing in the form of wear and fracture processes. As a result, the leaf after testing contains a greater number of particles, but these appear to be of smaller size and more equiaxed than the original particles.

## 4. Discussion

This study introduces a novel experimental methodology based on a combination of nanotechnology techniques and micromechanical/materials testing to simulate, for the first time, *in vitro* microwear of teeth caused by sliding contacts of soft matter containing abrasive particles. The artificial leaves employed have thickness and stiffness comparable to those of the leaves in botanical herbs and grasses. The new methodology enables investigations into aspects relevant to the mechanical degradation of teeth that cannot be ascertained by current standardized engineering tests and chewing simulators. Its feasibility was demonstrated in assessing the preliminary effects of artificial leaves, containing actual plant phytoliths, on human enamel wear. While tooth degradation by plant phytoliths is more relevant to other animal species (e.g. ungulates), human teeth provided controllable specimens, which helped simplify the analyses. It was concluded that, cumulatively, the presence of phytoliths in artificial leaves amplifies the extent of any pre-existing wear on the enamel surface, with a more pronounced effect as the initial damage becomes more severe.

The mechanism primarily responsible for wear is attributed to ‘plastic’ deformation caused by the sharper edges of individual phytoliths indenting and scratching the enamel [6]. The characteristic plastic grooves (microscratches) observed have different widths, resulting from the different attack angles of individual particles randomly distributed in the artificial leaf [10]. It is important to clarify that in the context of human enamel, ‘plastic’ denotes *permanent* deformation, achieved not through dislocation activity as in typical engineering materials but rather through the rupture of weak interphases, resulting in relative displacement (shear) of the mineral phase (i.e. quasi-plasticity; [34–38]). This is consistent with the general absence of pile-ups at the edge of the grooves. It is also consistent with measurements by other authors of enamel yield stresses under 1 GPa (e.g. [34,38,39]), well below the *H*/3 limit typical of dislocation plasticity [40].

There is evidence of the quasi-plastic mechanism operating both at the microstructural scale, involving the relative shear of enamel rods, and at the nanoscale, encompassing the relative shear of nanocrystals within rods. The microstructural scale is anticipated to dominate the deformation behaviour, given its potential for greater displacements. Evidence of inter-rod sliding can be found in the cross-sectional profiles of microscratches that intercept rod interfaces, as failures within the protein-rich sheathes facilitate relative sliding between neighbouring rods, forming characteristic steps (figure 5G). A scaled-down version of these shear processes operates at the nanoscale within individual rods, resulting in the distortion of the rod structure observed on the surface (figure 6C). In this context, the frictional dissipation associated with the relative sliding of nanocrystals is deemed responsible for the observed loss of mineral content upon prolonged leaf-enamel contacts [33,41].^1^ It is worth noting that, while interfacial failure is largely considered to be the key element of the inelastic deformation of enamel [34–38], recent work has brought this assumption into question [42], and further fundamental research is necessary.

Based on the identified microwear mechanisms, if particles remain sharp, the cumulative wear rates induced by artificial leaves could potentially reach severe macrowear conditions according to the tribology/engineering gradation (approx. 10^–6^ mm^3^ (N m)^−1^; [43]). However, even for relatively large biting forces, such wear rates would be compatible with durabilities well-adjusted to mammal lifetime [10]. The identified loss of mineral content upon prolonged contacts can result in a decrease in the hardness of the surface of outer enamel, which, according to Archard’s law, has the potential to compromise the long-term resistance to abrasion [16].

The effects on tooth enamel of phytoliths present in soft matter are consistent with what was observed in a previous study employing a hard zirconia antagonist [24]. In that work, phytoliths were dispersed as third bodies in (liquid) artificial saliva and were found to cumulatively enhance wear damage compared with tests without particles. However, a crucial difference is that phytoliths in an artificial leaf medium undergo significant mechanical degradation during testing—as is also observed in tests using individual particles [6]—while phytoliths in the artificial saliva/zirconia media remained largely unchanged at the conclusion of the tests. This effect can be explained based on differences in load transfer due to the stiffness of the phytoliths relative to the media [44]. A zirconia countersphere (elastic modulus of approx. 200 GPa) is significantly more rigid than opaline phytoliths and thus would shield a large part of the contact stress from the particles, which are more likely to remain structurally sound. By contrast, in an artificial leaf, the resin has a lower elastic modulus than the phytoliths. As a result, phytoliths would bear a greater fraction of the contact stress, thus increasing the likelihood of particle fracture/wear.

These results have implications for our understanding of the dietary adaptations of living and extinct animals. Different species have independently evolved strategies for dealing with heavy tooth wear. These strategies include taller teeth (hypsodonty), ever-growing teeth (hypselodonty) and thicker enamel, as well as behavioural modifications to the chewing cycle [45,46]. However, there is still significant debate about the cause of that wear. The confirmation of phytoliths as agents of tooth wear thus informs our reconstructions of dietary evolution and adaptation (e.g. [23,47,48]).

In biological anthropology, the field of dental microwear analysis has contributed substantially to our understanding of dietary evolution. Dental microwear analysis (e.g. [9,11]) and dental microwear texture analysis (e.g. [49,50]) aim at the quantification of microscopic wear on the dental surface (usually, the enamel surface). By quantifying patterns of scratches or pits or the surface texture using fractal analysis or tribological parameters, researchers can reveal aspects of an animal’s dietary ecology. For example, by studying the microscopic wear on the teeth of dead or extinct animals, researchers can estimate aspects of an animal’s diet, such as whether the animal consumed hard or soft foods, or whether the animal consumed fruits or plant matter, like leaves and grasses. By examining how microscopic dental wear has changed over recent times, researchers can track changes in a species’ diet. This study confirms that phytoliths in plant material are able to contribute to the microwear signal and influence our interpretation of diet.

Studies investigating how tooth microwear may contribute to tooth failure have had conflicting results. Comparative, longitudinal *in vivo* studies have found that microscopic scratches on the enamel’s surface are lost more easily than pits [11,51]. Dental microwear can be erased so quickly, particularly in an abrasive diet, that it is widely recognized that the microscopic wear can sometimes only reflect the most recent history of foods consumed by the animal (termed the ‘last supper’ effect; [11]). Laboratory studies by Xia *et al.* [23] and Lucas *et al.* [6] examined the mechanisms of dental microwear, both concluding that phytoliths could and could not cause microscopic wear; the latter study instead claimed phytoliths only cause plastic deformation, but not enamel removal. The former study witnessed similar effects, but—after running the samples under de-ionized water—saw the displaced enamel wash away. Consistent with results from this study, they concluded that phytoliths do, in fact, remove enamel. The microcontact load (i.e. the load at individual particle–enamel contacts) has been proposed as a key parameter that can reconcile the seemingly conflicting results from single-phytolith tests: a threshold load is defined at the onset of microcracking, with sub-threshold microcontact loads not being able to cause significant wear [24].

*In vivo* animal models investigating dental microwear/dental microwear texture formation have contributed substantially to our understanding of dental mechanical failure, enabling the application of realistic masticatory kinematics in a natural environment. *In vivo* feeding studies, carried out on both domesticated (e.g. rabbits, guinea pigs, goats) and wild laboratory (e.g. monkeys) animals, have investigated the effects of food composition and structural properties (e.g. qualitative measures of hardness/brittleness). For example, experiments on rabbits fed pellets consisting of lucerne, lucerne and oats, grass and oats or grass found that lucerne, which had relatively lower levels of silica, formed more pits while grass, which had relatively higher levels of silica, formed more scratches. The authors concluded that, contrary to prior interpretations of dental microwear texture, ‘A high variability in microwear and texture analysis thus need not represent dietary diversity, but can also be related to a uniform, low-abrasion diet’ [52]. Similar experiments on goats [53] fed pellets of lucerne (low abrasion, phytolith), grass (medium abrasion, phytolith), grass and rice husk (high abrasion, phytolith) and grass with sand (extremely abrasive) found differing levels of phytoliths caused different gradients in dental microwear texture, and there was no consistent pattern found in upper and lower molars, indicating the addition of phytoliths in diet can cause unpredictable changes in dental microwear texture. When investigating the effect of food structural properties in capuchins, Teaford and colleagues found monkeys fed only unshelled Brazilian nuts (representing hard food items) had more new microwear features (scratches/pits) than when they were fed a mixture of foods, including nuts and dried fruits [54]. Together, these studies imply that phytoliths can cause microwear damage to enamel, but the concentration of phytoliths and matrix within which microscopic damaging particles are embedded can affect the formation of dental microwear features.

Tooth *fracture* has also been used to reconstruct aspects of diet. Constantino *et al.* [15,55,56] have used chipping fractures to estimate the maximum bite force of extinct and extant mammals, including fossil hominins. Towle *et al.* [57–59] have similarly used the patterning of enamel fracture across dental rows to reconstruct the diets of extinct animals. The results of the current study impact these earlier works because they imply that phytoliths, when compressed with a high enough force into the dental enamel, could cause small fractures like those measured in these studies.

It is important to note the potential that the developed methodology holds for future studies, with some caveats. Firstly, it enables investigations into the effects of specific mechanical parameters (chewing force, number and speed of cycles, etc.), which can be easily modified during micromechanical tests. However, achieving quantitative analyses with statistical significance will necessitate conducting a more extensive number of tests on larger sets of enamel specimens than were conducted in the present study. Additionally, the arrangement of phytoliths in the model leaf may differ from natural conditions, potentially affecting their interaction with enamel and the formation of dental microwear features. Furthermore, the medium used to hold the phytoliths does not have the same structure as a leaf (e.g. lacking veins) and may degrade differently. Finally, the proposed methodology opens the door to developing even softer media capable of simulating the effects on enamel wear caused by soft tissues (such as the tongue) and food bolus—factors often overlooked in *in vitro* studies employing chewing simulators.

## 5. Conclusions

We have developed a novel experimental methodology based on nanotechnology techniques and micromechanical/materials testing to simulate and characterize microwear caused by the sliding of soft matter containing abrasive particles against human enamel. We found:

1. Plant phytoliths, upon cyclic contacts, increase the extent of pre-existing wear in tooth enamel and decrease its mineral content.
2. The primary wear mechanism of enamel is quasi-plastic deformation enabled by failure of weak interphases between—and within—mineral rods.
3. Phytoliths in soft solid media undergo long-term mechanical degradation.

These results provide a better understanding of how mechanical failure of dental enamel (microscopic wear, fracture, etc.) occurs, which has large implications for dentistry and understanding animal ecology and evolution.

## Data Availability

The datasets used in the analysis and the raw measurement data are available from Zenodo [60]. Supplementary material is available online [61].

## Footnotes

^1^
Under applied stress, the soft protein phase within enamel rods undergoes large shear deformations [34,35,38]. Its thinning or failure brings hydroxyapatite crystals into closer contact, with attendant increase of friction upon relative sliding [34,41].
